# Erratum: Cingulate Cortex Atrophy Is Associated With Hearing Loss in Presbycusis With Cochlear Amplifier Dysfunction

**DOI:** 10.3389/fnagi.2019.00131

**Published:** 2019-06-12

**Authors:** Frontiers Production Office

**Affiliations:** Frontiers Media SA, Lausanne, Switzerland

**Keywords:** presbycusis, neurodegeneration, dementia, cognition, cochlear amplifier

Due to a production error, Table 4 was erroneously typeset, thereby three rows were deleted. The correct Table is provided below. The publisher apologizes for this mistake. The original article has been updated.

**Table 4:** Pearson partial correlation corrected by age, sex, and education between cognitive tests and volume and thickness of anterior and posterior cingulate cortex for PCF and CD groups (^*^p < 0.05).

**Cognitive tests**	**ACC volume**				**ACC thickness**				**PCC volume**				**PCC thickness**			
	**PCF**		**CD**		**PCF**		**CD**		**PCF**		**CD**		**PCF**		**CD**	
	**r-Left**	**r-Right**	**r-Left**	**r-Right**	**r-Left**	**r-Right**	**r-Left**	**r-Right**	**r-Left**	**r-Right**	**r-Left**	**r-Right**	**r-Left**	**r-Right**	**r-Left**	**r-Right**
Cognitive functions (MMSE)	0.14	0.46^*^	−0.19	0.00	0.22	0.17	0.03	−0.13	0.05	0.40	−0.10	0.00	0.14	0.14	−0.01	−0.19
Executive functions (FAB)	0.29	0.07	−0.01	0.21	0.14	0.26	0.62	0.13	−0.12	−0.13	−0.01	0.02	−0.03	0.04	−0.11	−0.24
Visuospatial capacities (Rey figure)	0.05	0.41^*^	0.14	0.30	0.01	0.28	0.15	0.49^*^	0.32	0.27	0.35	0.54^*^	0.00	0.02	0.35	0.45^*^
Processing speed (TMT A)	−0.27	0.01	−0.37	−0.04	−0.22	−0.17	−0.34^*^	−0.26	−0.40^*^	−0.21	−0.41^*^	−0.31	0.02	−0.01	−0.20	−0.11
Episodic Memory (FCSRT)	−0.20	0.19	0.44^*^	0.31	0.01	0.08	0.34	0.10	−0.26	0.05	0.28	0.22	0.05	0.11	0.08	−0.18
Nomination (Boston)	0.21	0.03	0.47^*^	0.29	0.03	0.07	0.48^*^	0.41^*^	0.19	0.11	0.23	0.31	−0.16	0.03	0.28	0.20
Working memory (backward digit n°)	0.10	−0.14	0.64^*^	0.40	−0.06	−0.42	0.18	0.07	0.10	0.05	0.45^*^	0.18	0.12	0.40	−0.35	0.00
Depression score (GDS-15)	0.32	−0.20	−0.53^*^	−0.01	−0.17	0.36	0.05	0.00	0.17	0.00	−0.12	0.04	0.27	0.35	0.25	−0.09

