# Supplementary material for: Cingulate Cortex Atrophy Is Associated With Hearing Loss in Presbycusis With Cochlear Amplifier Dysfunction
Source: Front Aging Neurosci. 2019 Apr 26;11:97. doi: 10.3389/fnagi.2019.00097 (PMC6497796; doi:10.3389/fnagi.2019.00097)
Supplement: Supplementary file 1 [file Data_Sheet_1.PDF]

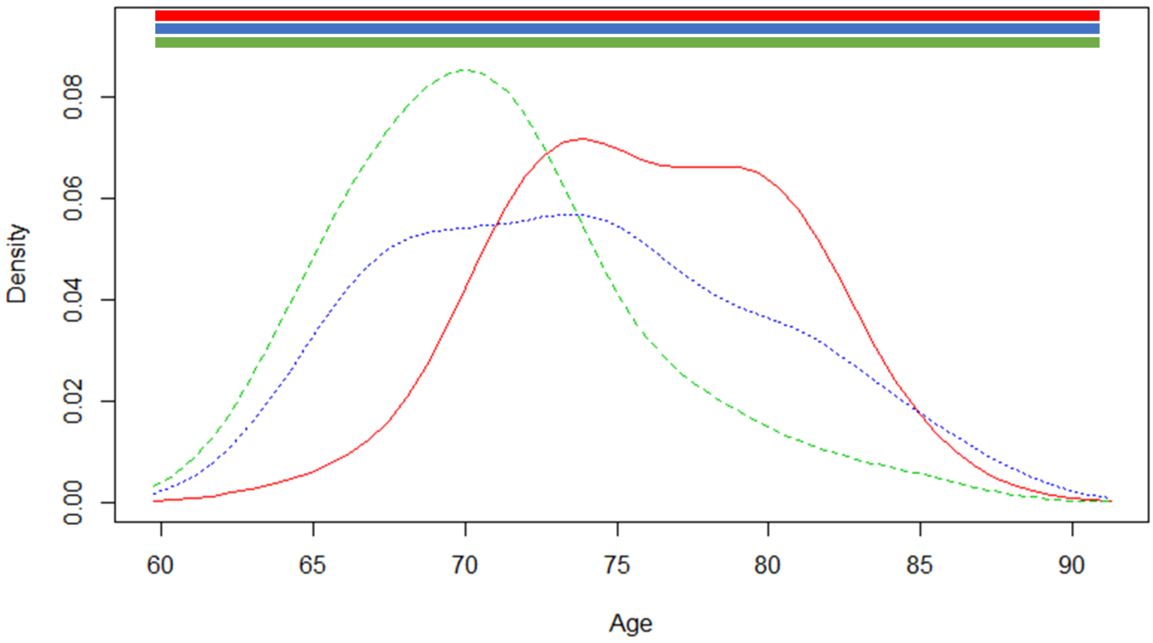

Supplementary material 1: Density plots for age (showing the distribution of probabilities of finding a participant with a particular age) present the differences between the age of the three groups; Control (Green), Presbycusis with preserved cochlear function (Blue), and Presbycusis with cochlear dysfunction (red). Bars above the density plot shows the range of the distributions per group using the same color labeling. As it can be observed, all three groups share the same age range, where central measures of average, median and modes differ. The control group presents the most different distribution, while there were no statistically significant differences between the two groups of presbycusis. (Presbycusis and Presbycusis with cochlear dysfunction present a highly overlapped distribution.

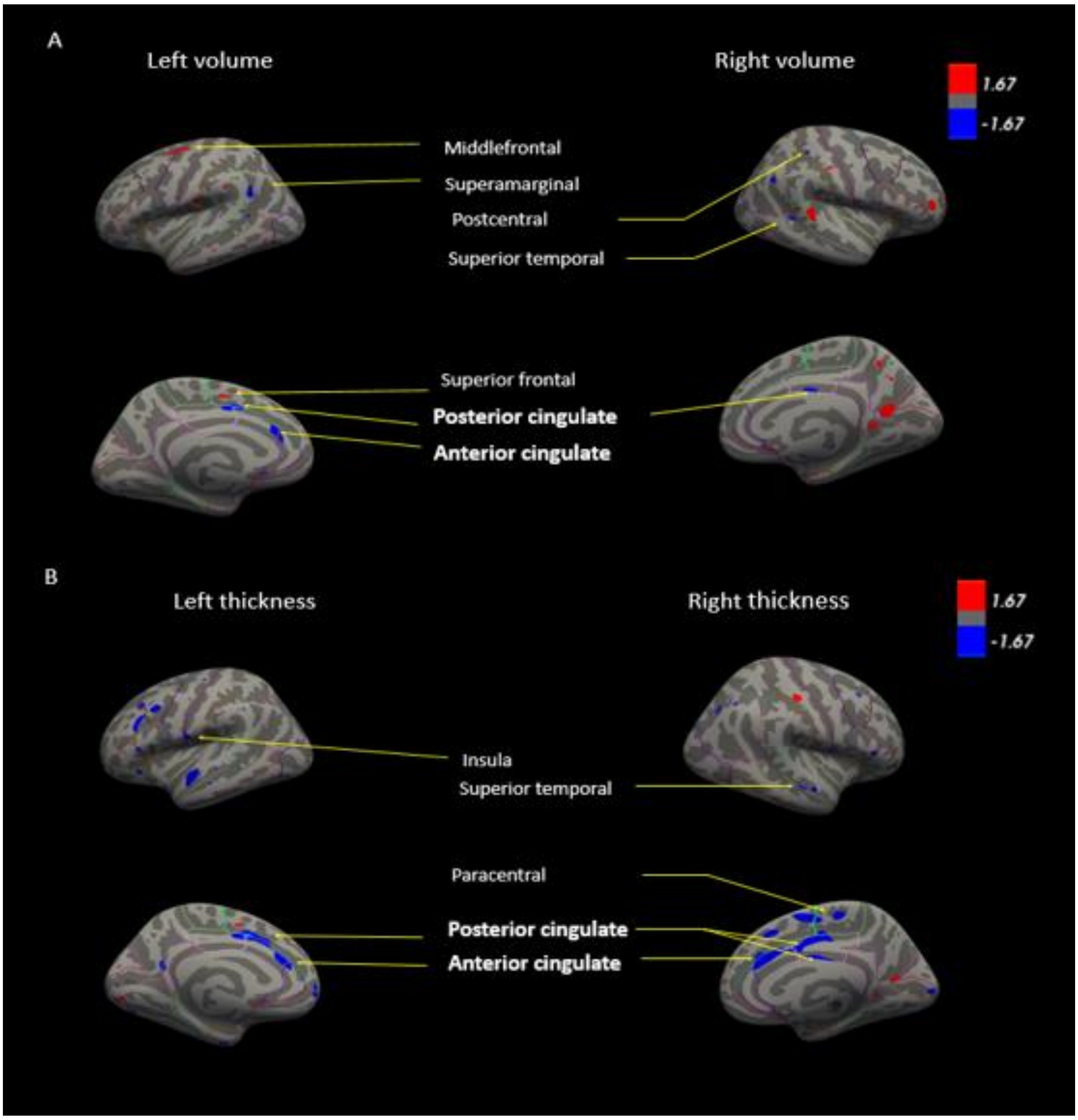

Supplementary material 2: GLM models for brain volume (A) and cortical thickness (B) correlated with PTA in presbycusis patients. We re-ran the GLM analysis while removing four patients whose PTA are over 45dB. The GLM contrasts the difference of correlations between PCF and CD groups using PTA as predictor for both volume and thickness. Age, education and sex were adjusted as covariates. The color blue shows regions in which individuals with cochlear dysfunction had a higher rate of gray matter decrease compared to those with normal cochlear ($p< 0.01$). The color red shows regions in which individuals with cochlear impairment had a higher rate of gray matter increase compared to those with normal cochlear ($p <0.01$). The anterior and posterior cingulate atrophy did not show any significant differences compared with our previous data presented in the manuscript. This result confirms that brain changes are due to cochlear dysfunction and not to hearing loss effect.
